# Four New Chloro-Eremophilane Sesquiterpenes from an Antarctic Deep-Sea Derived Fungus, *Penicillium* sp. PR19N-1

**DOI:** 10.3390/md11041399

**Published:** 2013-04-23

**Authors:** Guangwei Wu, Aiqun Lin, Qianqun Gu, Tianjiao Zhu, Dehai Li

**Affiliations:** Key Laboratory of Marine Drugs, Chinese Ministry of Education, School of Medicine and Pharmacy, Ocean University of China, Qingdao 266003, China; E-Mails: gweiwu@163.com (G.W.); linaiqun@yahoo.cn (A.L.); guqianq@ouc.edu.cn (Q.G.)

**Keywords:** chlorinated eremophilane sesquiterpene, Antarctic deep-sea derived fungus, *Penicillium* sp.

## Abstract

A new chloro-trinoreremophilane sesquiterpene **1**, three new chlorinated eremophilane sesquiterpenes **2**–**4**, together with a known compound, eremofortine C (**5**), were isolated from an Antarctic deep-sea derived fungus, *Penicillium* sp. PR19N-1. Structures were established using IR, HRMS, 1D and 2D NMR techniques. In addition, the plausible metabolic network of these isolated products is proposed. Compound **1** showed moderate cytotoxic activity against HL-60 and A549 cancer cell lines.

## 1. Introduction

**Figure 1:**
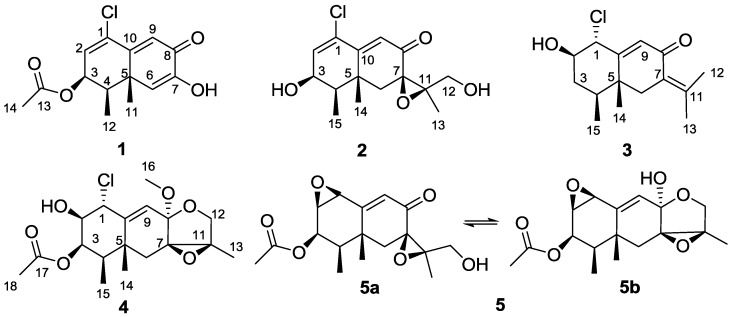
Structures of compounds **1**–**5**.

The Antarctic Ocean is inherently considered a harsh habitat for native microorganisms due to perpetual low temperatures and lack of nutrients, among other factors [1,2]. However, in the case of fungi inhabiting the Antarctic deep sea, the aforementioned extreme conditions set the expression of unusual biosynthetic mechanisms that may lead to unique secondary metabolites [3]. Undeniably, the exploitation of these peculiar metabolic pathways represents a new opportunity for the discovery of bioactive secondary metabolites [4]. Thus, the research community has been urged to explore the untapped metabolic reservoir originating from deep-sea fungi in order to combat human diseases [5]. In our efforts to search for novel active compounds from the secondary metabolites of deep-sea derived microorganisms [6,7,8], a fungus, identified as *Penicillium* sp. PR19N-1, was obtained from a deep-sea sediment collected in Prydz Bay (−1000 m). Its extract exhibited brine shrimp lethality activity. Studies on the active constituents of this fungus led to the isolation of four new chlorinated eremophilane sesquiterpenes **1**–**4**, along with a known compound, eremofortine C (**5**) [9,10] (Figure 1). Herein, we describe their isolation, structure elucidation and in vitro cytotoxicity evaluation.

## 2. Results and Discussion

**Figure 2:**
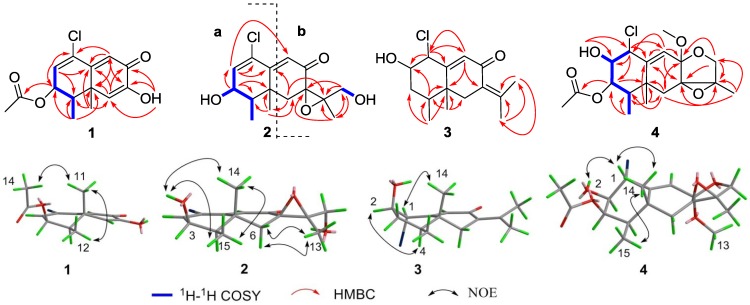
The key 2D NMR correlations for compounds **1**–**4**.

Compound **1** was obtained as an optically active colorless oil. The molecular formula of C_14_H_15_ClO_4_ was established through HRESIMS data ([M + Na]^+^ 305.0543, calcd. 305.0557), indicating seven double bond equivalents. The IR spectrum showed absorption bands characteristic for hydroxyl, carbonyl and double bond moieties at 3292, 1731, 1634 cm^−1^, respectively. One-dimensional NMR data (Table 1, Table 2) unveiled 8 sp^2^ deshielded carbons (1 × OC=O, 3 × CH=C, 1 × C=O), and 6 sp^3^ shielded carbons (3 × CH_3_, 2 × CH, 1 × C), indicating the presence of two rings in the molecule. The two fused six-membered rings were defined by extensive analysis of HMBC cross peaks from the diagnostic methyls H_3_-11 to C-4, C-5, C-6 and C-10, H_3_-12 to C-3, C-4, C-5, as well as from the olefinic protons H-2 to C-1, C-3, C-4, and C-10, H-6 to C-4, C-5, C-8, C-10, and C-11, and H-9 to C-1, C-5, C-7, and C-10. Extensive analysis of MS and NMR data led us to a trinor-eremophilene core with an 8-oxo-1(2),9(10)-diene unit [11,12]. The hydroxyl group attached to C-7 was positioned using HMBC correlations (Figure 2) between the exchangeable proton (OH-7) and C-6, C-7 and C-8. In addition, an acetoxy group was assigned to C-3 via HMBC correlations between H-14 and C-13, and between H-3 and C-13. Furthermore, the COSY-defined spin system H-2/H-3/H-4/H_3_-12 along with the lack of an olefinic proton signal at C-1 in the HMQC spectrum indicated the location of a chlorine atom at C-1.

**Table 1:** ^13^C NMR data for compounds **1**–**4** (150 MHz, δ ppm).

No.	1 ^a^	2 ^b^	3 ^a^	4 ^b^
1	133.5 s	129.5 s	67.6 d	63.1 d
2	129.1 d	138.4 d	74.9 d	75.3 d
3	70.3 d	66.9 d	37.1 t	75.6 d
4	37.7 d	39.8 d	38.5 d	38.6 d
5	42.7 s	38.5 s	42.8 s	42.2 s
6	121.3 d	40.3 t	41.1 t	32.0 t
7	146.3 s	61.8 s	126.6 s	67.3 s
8	181.1 s	194.9 s	191.1 s	100.6 s
9	123.2 d	125.3 d	127.9 d	122.0 d
10	159.7 s	157.3 s	159.9 s	144.4 s
11	23.7 q	67.3 s	144.3 s	65.3 s
12	11.5 q	61.2 t	22.6 q	68.7 t
13	170.4 s	16.1 q	22.2 q	12.7 q
14	20.9 q	23.6 q	16.9 q	23.3 q
15		11.0 q	14.9 q	11.2 q
16				50.9 q
17				170.0 s
18				20.8 q

^a^ Resolved in CDCl_3_. ^b^ Resolved in DMSO.

**Table 2:** ^1^H NMR data for compounds **1**–**4** (600 MHz, δ ppm, *J* in Hz).

No.	1 ^a^	2 ^b^	3 ^a^	4 ^b^
1			4.48, dd (10.0, 2.0)	4.72, dd (10.5, 1.3)
2	6.36, d (5.0)	6.63, d (6.4)	3.63, m (10.0, 9.4, 5.0)	3.36, ddd (10.5, 7.3, 3.7)
3	5.50, dd (5.0, 5.0)	4.14, ddd (6.4, 5.3, 4.2)	1.61, m	5.23, dd (3.7, 3.7)
4	2.12, m	1.80, qd (7.0, 4.2)	2.04, m	1.74, qd (6.9, 3.7)
6	6.28, s	a: 2.33, d (14.6)	a: 2.88, d (13.7)	a: 1.89, d (14.4);
		b: 1.89, d (15.1)	b: 2.26, d (13.7)	b: 1.83, d (14.4)
9	6.76, s	6.28, s	6.41, d (2.0)	5.94, d (1.3)
11	1.42, s			
12	1.18, d (6.8)	3.70, dd (6.0, 5.8)	2.11, s	a: 3.89, d (10.4);
				b: 3.53, d (10.4)
13		1.39, s	1.87, s	1.35, s
14	2.13, s	1.20, brs	1.04, s	1.19, s
15		1.04, d (7.0)	1.02, d (6.4)	0.84, d (6.9)
16				3.30, s
18				2.08, s
2-OH				5.69, d (7.3)
3-OH		5.27, d (5.3)		
7-OH	6.32, brs			
12-OH		4.81, t (5.8)		

^a^ Resolved in CDCl_3_. ^b^ Resolved in DMSO.

**Figure 3:**
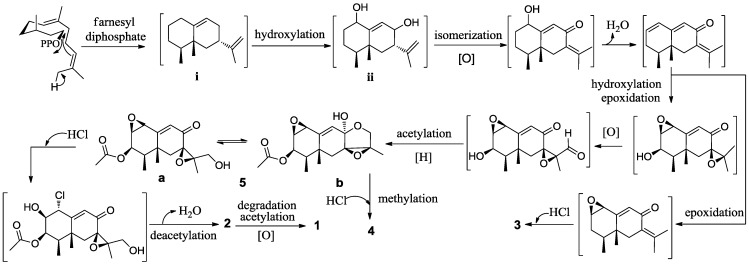
Proposed biogenetic network for compounds **1**–**5**.

The relative configuration of the trinor-eremophilane core was deduced on the basis of NOE-difference experiments (Figure 2). The resonances of H-12 and H-14 were notably enhanced as a result of irradiating CH_3_-11, indicating that the axial-methyl at C-5, the equatorial-methyl at C-4, and the acetyl group at C-3 were co-facial. The coupling constants (^3^*J*_H-2, __H-3_ = 5.0 Hz; ^3^*J*_H-3, __H-4_ = 5.0 Hz ) in **1** similar with related data in known compounds that possessed the same partial structure [11,13] further confirmed the relative configuration at C-3 as β*-*orientation. The same relative configuration for CH_3_-11 and CH_3_-12 can also be inferred from a biogenetic pathway (Figure 3) involving the trans-conformation of the decalin unit of eremophilane sesquiterpenes and comparison with previously reported congeners [14,15,16]. Thus, **1** was determined as 1-chloro-3β-acetoxy-7-hydroxy-trinoreremophil-1,6,9-trien-8-one.

The molecular formula of the monochlorinated compound **2** was established as C_15_H_19_ClO_4_ on the basis of an HRESIMS parent ion [M + H]^+^ at *m/z* 299.1060 (calcd. 299.1050). Key ^1^H and ^13^C NMR resonances (Table 1, Table 2), especially for the shielded methyl groups at δ_H_ 1.04 (*J* = 7.0 Hz) and δ_H_ 1.20, led us to consider an eremophilane-type sesquiterpene skeleton for **2**. The presence of an epoxide moiety with ^13^C peaks at C-7 (δ_C_ 61.8) and C-11 (δ_C_ 67.3) was suggested by comparison of the ^13^C NMR spectrum with those of **5a** [9,10], and was confirmed by HMBC correlations (Figure 2) from H-13 to C-7, C-11 and C-12, from H_2_-6 to C-7, C-10, C-11 and C-14, and from H-12 to C-7, C-11 and C-13. According to the ^1^H–^1^H COSY correlation between 12-OH and H_2_-12, the sole primary alcohol was also located at C-12. Thus, the above evidence suggested **2** and **5a** had the same substructure **b** (Figure 1) [9,10]. Careful analysis of the NMR data of **2** indicated the ring A was similar to that in compound **1**. The main differences of them were the 3-acetoxy group replaced by 3-OH which was confirmed by COSY correlations of H_3_-15/H-4/H-3 (Figure 2) and chemical shift at C-3 (δ_C_ 66.9) in **2**. The *cis* configuration of H-3 and H-4 was assigned according to their shared coupling constant (^3^*J*_H-3, H-4_ = 4.2 Hz), which indicated the β-orientation of 3-OH, conformed by the NOESY correlation between OH-3 and Me-14 and Me-15. The β-orientation of the epoxide at C-7 and C-11 in **2** was evidenced by the NOE effects from Me-13 to both H-6a and H-6b, as well as the similar NMR data at C-6, C-7, C-11, C-12 and C-13 between **2** and **5a**.

Compound **3** was obtained as optically active colorless oil. The LRESIMS showed an [M + H]^+^ peak at *m/z* 269/271 (rel. 3:1), and the HRESIMS led to the molecular formula C_15_H_21_ClO_2_ (exp. 269.1312, calcd. 269.1308), consistent with five degrees of unsaturation. The IR spectrum showed the presence of hydroxyl, carbonyl and double bond moieties, displaying characteristic absorption bands at 3418, 1715, 1656 cm^−1^, respectively. The ^1^H and ^13^C NMR resonances (Table 1, Table 2) of **3** were reminiscent of the known eremophilane-type sesquiterpenes that possessed an 8-oxo-7(11),9(10)-diene units [17,18]. The related HMBC correlations were further confirmed as shown in Figure 2. The remaining chlorine and hydroxyl groups were assigned to C-1 and C-2 respectively, on the basis of chemical shift values for C-1 (δ_C_ 67.6) and C-2 (δ_C_ 74.9, as well as key HMBC correlations of H-1with C-2, C-9 and C-10 (Figure 2). 

The relative configuration of **3** was determined using NOE-difference experiments and coupling constant analysis. The anti configuration of H-1 and H-2 was given by their common coupling constant (^3^*J*_H-1, __H-2_ = 10.0 Hz). NOE enhancements from H-1 to Me-14 and from H-2 to H-4 indicated a co-facial relationship for H-1, OH-2, CH_3_-14 and CH_3_-15. Thus, compound **3** was elucidated as 1α-chloro-2β-hydroxyeremophil-7(11),9-dien-8-one.

LRESIMS peaks of [M + Na]^+^ parent ion at *m/z* 395/397 (rel. 3:1) and the corresponding HRESIMS value at *m/z* 395.1238 [M + Na]^+^ supported the molecular formula of **4** as C_18_H_25_O_6_Cl (calcd. 395.1237). In ^1^H NMR spectrum, two typical eremophilane-type methyls [δ_H_ 1.19; 0.84 (*J* = 6.9 Hz)], three downfield protons (δ_H_ 4.72, 3.36, 5.23), a methoxyl group (δ_H_ 3.30) were observed. Eighteen carbon signals were distributed into five methyls (including one methoxyl), two methylenes, five methines, six quaternary carbons in the ^13^C NMR spectrum. Extensive analysis of NMR data of **4** indicated that the structure of **4** was similar with that of **5b** [9,10], except for the 8-OCH_3_, 1-Cl and 2-OH group in **4** instead of 8-OH and 1,2-epoxide group in **5b**. Detailed HMBC correlations (Figure 2) further supported the above result. HMBC correlations from CH_3_-15 to C-3 and C-4, as well as ^1^H–^1^H COSY connectivities of H_3_-15 with H-4, and H-4 with H-3, deduced C-3 as an oxygenated methine. The acetyl group was located at C-3 by HMBC correlation of H-3 with C-17. ^1^H–^1^H COSY correlations between OH-2 and H-2, and HMBC correlations from the exchangeable proton (2-OH) to C-1 and C-2 determined hydroxyl group at C-2. The gross structure of **4** was completed by assigning the chlorine atom to C-1. NOESY cross-peaks between H-1, H-14 and OH-2 indicated the relative configuration to be 1α-Cl and 2β-OH, respectively (Figure 2). The small coupling constant between H-3 and H-4 (^3^*J*_H-3,__ H-4_ = 3.7 Hz) indicated the relative configuration of H-3 was α-orientation. The relative stereochemistry of 8-OCH_3_ and epoxide group at C-7 could be deduced as α and β-orientations, respectively on the basis of biosynthetic origin with **2** and stereochemistry of tautomer **5a** and **5b**.

The formation of an eremophilane sesquiterpene core (i) could be traced back to the universal precursor farnesyl diphosphate, which is the substrate of cascade cyclization [19]. In subsequent modifications, the key intermediate 1,8-dihydroxy product (ii), likely generated via cytochrome P450 monooxygenases [20], could be modified through a series of standard biochemical transformations (isomerization, hydroxylation, carbonylation, epoxidation, cycloaddition, degradation, halogenation among other) to finally yield compounds **1**–**5** (Figure 3).

All new compounds **1**–**4** were evaluated for their cytotoxic activity against HL-60 using an MTT method [21] and A549 cancer cell lines using an SRB method [22]. Compound **1** show modest cytotoxic activity against HL-60 and A549 cell lines with IC_50_ values of 11.8 ± 0.2 and 12.2 ± 0.1 μM, respectively. The other tested compounds were not cytotoxic (IC_50_ > 50 μM) towards the above two cell lines.

## 3. Experimental Section

### 3.1. General Experimental Procedures

Specific rotations were obtained on a JASCO P-1020 digital polarimeter. UV spectra were recorded on Cary 300 spectrophotometer. IR spectra were recorded on a NICOLET NEXUS 470 spectrophotometer in KBr discs. ^1^H, ^13^C-NMR, DEPT and 2D-NMR spectra were recorded on a JEOL JNM-ECP 600 spectrometer using TMS as internal standard and chemical shifts were recorded as δ values. 1D NOE experiment was carried out by a Varian INOVA-400 spectrometer. Mass spectra were obtained on a Micromass Q-TOF ULTIMA GLOBAL GAA076 LC Mass spectrometer or on a GCT-MS Micromass UK Mass spectrometer. Semipreparative HPLC was performed using an ODS column (YMC-Pack ODS-A, 10 × 250 mm, 5 μm, 4 mL/min). Yeast extract and peptone were produced by Bingjing Shuangxuan Microbe Culture Medium Products Factory.

### 3.2. Fungal Material

The working strain *Penicillium* sp. PR19N-1 was isolated from marine sludge collected from Prydz Bay (−1000 m), Antarctica. The isolate was identified as *Penicillium* sp. according to morphological traitsr and ITS rDNA sequence analysis. The sequence data have been submitted to GenBank, accession number KC756948. The strain was deposited in the Key Laboratory of Marine Drugs, the Ministry of Education of China, School of Medicine and Pharmacy, Ocean University of China, Qingdao, China. Working strain was prepared on Potato Dextrose agar slants and stored at 4 °C, the accession number is MBC06294.

### 3.3. Fermentation and Extraction

The fungus was grown on rotary shaker (165 rpm) at 15 °C for 14 days in 500-mL conical flask containing the liquid medium (150 mL/flask) that was composed of yeast extract 3 g/L, maltose 3 g/L, glucose 20 g/L, peptone 5 g/L and sea salt (The main chemical composition is NaCl) 24.4 g/L after adjusting its pH to 7.2–7.4.

The fermented whole broth (50 L) was filtered through cheesecloth to separate into supernatant and mycelia. The supernatant was extracted three times with ethyl acetate, while the mycelia were extracted three times with 70% aqueous acetone. The acetone solution was concentrated under reduced pressure to afford an aqueous solution, which was extracted three times with ethyl acetate. Both ethyl acetate solutions were combined and concentrated under reduced pressure to give a crude extract (29.5 g).

### 3.4. Purification

The crude extract (29.5 g) was separated into seven fractions (F1–F7) subjected to vacuum liquid chromatography on a silica gel column using a step gradient elution with CHCl_3_–MeOH (100%–50%). Faction 4 showed the moderate cytotoxic activity. The fraction 4 was chromatographed on a Sephadex LH-20 column with CHCl_3_–MeOH (1:1) to produce four subfractions (4.1–4.4). The subfraction 4.3 was further chromatographed on a silica gel column using a step gradient elution of petroleum ether-acetone to give six fractions (4.3.1–4.3.6). Compound **1** (1.4 mg) was isolated from the subfraction 4.3.1 by semipreparative HPLC (60:40 MeOH–H_2_O, 4.0 mL/min). The subfraction 4.3.3, obtained from the elution of petroleum ether-acetone (15:1), was further applied to semipreparative HPLC (60:40 MeOH–H_2_O) to yield compounds **3** (1.3 mg). The subfraction 4.3.5, obtained from the elution of petroleum ether:acetone 5:1, was chromatographed on a Sephadex LH-20 column with MeOH to give six fractions (4.3.5.1–4.3.5.6). The subfraction 4.3.5.3 was isolated by semipreparative HPLC (40:60 MeOH–H_2_O, 4.0 mL/min) to yield compound **4** (7.2 mg). The subfraction 4.3.2 was further fractionated by semipreparative HPLC (50:50 MeOH–H_2_O, 4.0 mL/min) to yield compound **2** (2.1 mg) and subfraction 4.3.2.1, which was further purified by semipreparative HPLC (35:65 MeOH–H_2_O, 4.0 mL/min) to yield compound **5** (20.6 mg).

Compound **1**: colorless oil; [α]_D_^20^ +274 (*c* 0.05, CHCl_3_); UV (MeOH) λ_max_ (log ε) 223 (4.2), 277 (4.1) nm; IR (KBr) ν_max_ 3292, 1731, 1634, 1235, 1012, 918, 784 cm^−1^; ^1^H-NMR and ^13^C-NMR data, see Table 1, Table 2; HRESIMS *m/z* 305.0543 [M + Na]^+^ (calcd. for C_14_H_15_O_4_ClNa, 305.0557).

Compound **2**: colorless oil; [α]_D_^20^ +21 (*c* 0.1, CHCl_3_); UV (MeOH) λ_max_ (log ε) 255 (3.8), 279 (3.7, sh) nm; IR (KBr) ν_max_ 3455, 2966, 2939, 2886, 1733, 1670, 1455, 1260, 1100, 1023, 756 cm^−1^; ^1^H-NMR and ^13^C-NMR data, see Table 1, Table 2; HRESIMS *m/z* 299.1060 [M + H]^+^ (calcd. for C_15_H_20_O_4_Cl, 299.1050).

Compound **3**: colorless oil; [α]_D_^20^ +103 (*c* 0.1, CHCl_3_); UV (MeOH) λ_max_ (log ε) 247 (4.0), 280 (3.8, sh) nm; IR (KBr) ν_max_ 3418, 2988, 1715, 1656, 1620, 1436, 1365, 1272, 1171, 1088, 904 cm^−1^; ^1^H-NMR and ^13^C-NMR data, see Table 1, Table 2; HRESIMS *m/z* 269.1312 [M + H]^+^ (calcd. for C_15_H_22_O_2_Cl, 269.1308).

Compound **4**: colorless oil; [α]_D_^20^ −52 (*c* 0.05, CHCl_3_); UV (MeOH) λ_max_ (log ε) 200 (3.8) nm; IR (KBr) ν_max_ 3435, 3001, 2950, 2871, 1743, 1466, 1233, 1028, 995 cm^−1^; ^1^H-NMR and ^13^C-NMR data, see Table 1, Table 2; HRESIMS *m/z* 395.1238 [M + Na]^+^ (calcd. for C_18_H_25_O_6_ClNa, 395.1237).

### 3.5. Biological Assays

In the MTT assay, the cell lines were grown in RPMI-1640 supplemented with 10% FBS under a humidified atmosphere of 5% CO_2_ and 95% air at 37 °C. The amount of 200 μL of those cell suspensions at a density of 5 × 104 cell mL^−1^ was plated in 96 well microtiter plates and incubated for 24 h at the above given conditions. Then 2 μL of the test compound solutions (in MeOH) at different concentrations were added to each well and further incubated for 72 h under the same condition. Twenty μL of the MTT solution (5 mg/mL in IPMI-1640 medium) was added to each well and incubated for 4 h. An old medium (150 μL ) containing MTT was then gently replaced by DMSO and pipetted to dissolve any formazan crystals formed. Absorbance was then determined on a SPECTRA MAX PLUS plate reader at 540 nm.

In the SRB assay, 200 μL of the cell suspensions were plated in 96-cell plates at a density of 2 × 10^5^ cell mL^−1^. Then 2 μL of the test compound solutions (in MeOH) at different concentrations was added to each well and the culture was further incubated for 24 h. Following drug exposure, the cells were fixed with 10% trichloroacetic acid and the cell layer was stained with 0.4% SRB. The absorbance of SRB solution was measured at 520 nm. Dose response curves were generated and the IC_50_ values, the concentration of compound required to inhibit cell proliferation by 50%, were calculated from the linear portion of log dose response curves.

## 4. Conclusions

Chemical investigation of an Antarctic deep sea-derived fungus *Penicillium* sp. PR19N-1 has led to a new cytotoxic chloro-trinoreremophilane sesquiterpene **1**, three new chlorinated eremophilane-type sesquiterpenes **2**–**4**, along with a known compound, eremofortine C (**5**).

Eremophilane-type sesquiterpenes as a subclass of sesquiterpenoids have gained considerable attentions due to diverse structures and a broad range of biological activities [23,24], most of them distributed in terrestrial plants or microorganisms. To date, more than 900 eremophilane-type sesquiterpenes have been discovered, and to the best of our knowledge, it is the first example that chloro-eremophilane sesquiterpenes were found from microorganisms in the past 30 years.
